# Interplay Between Virulence and Variability Factors as a Potential Driver of Invasive Meningococcal Disease

**DOI:** 10.1016/j.csbj.2018.02.002

**Published:** 2018-02-15

**Authors:** Emilio Siena, Margherita Bodini, Duccio Medini

**Affiliations:** GSK, 53100 Siena, Italy

## Abstract

Neisseria meningitidis (Nm) is frequently found in the upper respiratory tract of the human population. Despite its prevalence as a commensal organism, Nm can occasionally invade the pharyngeal mucosal epithelium causing septicemia and life-threatening disease. A number of studies have tried to identify factors that are responsible for the onset of a virulent phenotype. Despite this however, we still miss clear causative elements. Several factors have been identified to be associated to an increased susceptibility to meningococcal disease in humans. None of them, however, could unambiguously discriminate healthy carrier from infected individuals. Similarly, comparative studies of virulent and apathogenic strains failed to identify virulence factors that could explain the emergence of the pathogenic phenotype. In line with this, a recent study of within host evolution found that Nm accumulates genomic changes during the asymptomatic carriage phase and that these are likely to contribute to the shift to a pathogenic phenotype. These results suggest that the presence of virulence factors in the meningococcal genome is not a sufficient condition for developing virulent traits, but is rather the ability to promote phenotypic variation, through the stochastic assortment of the repertoire of such factors, which could explain the occasional and unpredictable onset of IMD. Here, we present a series of argumentations supporting the hypothesis that invasive meningococcal disease comes as a result of the coexistence of bacterial virulence and variability factors in a plot that can be further complicated by additional latent factors, like host pre-existing immune status and genetic predisposition.

## 1 Introduction

*Neisseria meningitidis* (*Nm*) is a Gram-negative diplococcus that normally resides in the human nasopharynx in 8–25% of the worldwide population [1]. Despite its prevalence as a harmless, commensal organism, *Nm* can occasionally invade the pharyngeal mucosal epithelium causing septicemia and life-threatening disease. Many studies have tried to identify and understand the factors that are responsible for the onset of such a virulent phenotype [2,3]. Despite these efforts, however, we are still missing evidence for unambiguous causative elements.

The meningococcal carriage state is a result of the successful commensal relationship between the host and the bacterium and is likely to be influenced by additional latent factors like host's diet and microbiome composition. While living in this equilibrium state, *Nm* can be transmitted among susceptible individuals through direct contact or respiratory droplets. Under normal circumstances, *Nm* cells attempting to traverse the epithelial barrier to access the bloodstream are readily cleared by the host's immune defenses [4]. In those rare cases of immune evasion, however, the disease is fulminant developing within hours and leading to death if untreated within 2 days [5]; without inducing a shedding state in the diseased individual. Such bacterial cells are unlikely to be transmitted to new hosts, *de facto* running into an evolutionary dead end for *Nm*. Based on this notion, invasive meningococcal disease (IMD) has to be assumed as the result of a dysfunctional relationship with the host [6].

Investigations on factors interfering with the commensal relationship between *Nm* and its host, which could lead to the onset of IMD, have focused both on the host and on the pathogen side. Studies in humans have identified several genetic and immunological factors associated to the susceptibility to, and severity of meningococcal disease. These factors relate to the host's mucosal barrier, pattern recognition receptors of the innate immune system, antimicrobial peptides, proinflammatory mediators, components of the adaptive immune system, complement response and fibrinolysis. A comprehensive review is provided in Dale et al. [2]. However, none of those factors could unambiguously discriminate healthy carrier from infected individuals, meaning that host predisposition alone cannot fully explain *Nm* ability to cause disease.

Similarly, several *Nm* properties have been identified to be associated with an increased propensity to cause IMD. Among the 12 *Nm* serogroups characterized to date, only a subset (A, B, C, W, X and Y) have been typically associated with IMD, accounting alone for >90% of meningococcal invasive disease worldwide [7,8]. Epidemiological studies further identified specific genotypic lineages (clonal complexes) occurring with significantly higher frequency within IMD-causing than carriage isolates [3], suggesting that the ability to cause IMD is contributed by the specific genetic makeup of some *Nm* strains. Nonetheless, comparative studies of virulent and apathogenic strains failed to identify virulence factors (surface determinants and genes involved in host-pathogen interaction) that could unambiguously discriminate between the two phenotypes [9,10].

In line with this, a recent study investigating the evolution of *Nm* within the host, found that genomic changes, primarily affecting surface components involved in host-pathogen interaction, occur frequently in *Nm* during the asymptomatic carriage phase and that these are likely to contribute to the shift to a pathogenic phenotype [11,12]. Overall, collected evidences suggest that the presence of virulence factors in the meningococcal genome is not a sufficient condition for developing virulent traits but is rather the ability to promote phenotypic variation, through the stochastic assortment of the repertoire of such factors, which could explain the occasional and unpredictable onset of IMD.

A main driver of phenotypic variability in *Nm* is represented by Simple Sequence Repeats (SSRs), contiguous iterations of short DNA motifs that are highly prone to slipped strand mispairing during chromosome replication. Such unstable elements are capable to stochastically silent gene expression by introducing frameshifts in the reading frame or to modulate gene expression by altering the gene's transcriptional promoter [13,14]. Each *Nm* isolate carries on average 2000 genes and >4000 SSRs in its genome. A recent genomic analysis showed that 10 to 15% of *Nm* genes are possible targets of the regulation mediated by these repeats, with high frequency stochastic variation experimentally confirmed for 115 genes [15]. The extraordinary abundance of such variability hotspots has been described to be higher than what found in other prokaryotes [16] and in respect to random expectations [17], indicating that *Nm* relies on SSRs as a pivotal mechanism of evolution and rapid adaptation to fluctuating environmental conditions.

In this review, a series of argumentations will be presented supporting the hypothesis that IMD originates from the interplay between bacterial virulence and variability factors (chromosomic elements promoting high-frequency phenotypic switching).

## 2 Host Susceptibility to Meningococcal Disease

Despite the fact that meningococcal disease is predominant in individuals lacking preexisting immunity (defined as the presence of bactericidal antibodies in the plasma) to this pathogen, only a minority of these develop IMD [18]. Individuals lacking adaptive immunity against *Nm* necessarily rely on their innate immune system to prevent a systemic infection. Consequently, it was hypothesized that the apparently random onset of meningococcal disease could actually be due to host genetic factors, linked to the innate immune system, which may be associated to an increased susceptibility [2]. In line with this theory, a number of retrospective case-control studies identified multiple immune-related genes whose specific haplotypes, or polymorphisms, segregated susceptible and non-susceptible individuals with statistical significance. Specifically, altered susceptibility to meningococcal infection has been associated with specific alleles of genes coding for cell-surface receptors (CECAM3 and CECAM6) [19], pattern recognition receptors (TLR4 and TLR9) [20,21], complement pathway regulators (CFH and CFHR3) [22,23], antimicrobial peptides (DEFB1) [24] and pro-inflammatory cytokines (IL1RN and TNF-α) [25,26]. Robustness of these results, however, was generally hampered by underpowered sample sizes, inconsistency between patient inclusion criteria or failure to account for factors known to be associated with susceptibility. Consequently, some of the identified associations were weak [27,28] or could not be confirmed in independent validation cohorts [29] and further investigation is needed to clear up their truthfulness.

Despite the shortcomings, however, some innate immune genes involved in inflammatory response (IL1B, IL1RN and TNF [25,26]) and the complement cascade (CFH, CFHR3 [22,23]) were shown to have a pivotal role in host genetic predisposition to IMD. Complement factor H (CFH), for example, is a regulator of the complement pathway activation that function by either increasing the decay rate of the alternative pathway C3 convertase C3bBb or by acting as cofactor for Factor I mediated C3b cleavage. Remarkably, *Nm* has adapted to avoid complement-mediated killing by recruiting CFH molecules on its surface through the production of a CFH ligand called factor H binding protein (fHbp) [30]. Based on this evidence, it is postulated that high plasma levels of CFH can increase the chance of *Nm* survival in the blood, consequently leading to an increased susceptibility to meningococcal infection. Haralambous et al. [22] conducted a study to determine whether a single nucleotide polymorphism, located in the promoter region of the *CFH* gene (C to T conversion at position −496), has a role in IMD susceptibility. Genetic susceptibility was investigated in 2 independent studies, a case-control and family based transmission-disequilibrium-test, using 2 separate cohorts of UK Caucasian patients. A higher IMD susceptibility was found in patients homozygous for the C/C genotype [odds ratio (OR)  =  2.0, *p*  =  0.001]. Such association was even stronger for the cohort of patients infected with serogroup C isolates (OR  =  2.9, *p*  =  0.0002).

In conclusion, a number of genetic traits linked to IMD susceptibility have been identified that can be used as markers for increased, or reduced, chance to develop IMD or disease severity. Studies have also started providing mechanistic insights into IMD pathophysiology, like the pivotal role of the complement system in preventing meningococcal septicemia. However, the biology of the human interaction with his microbiota is complex and the analysis of individual factors is unlikely to tell the whole story about host predisposition to develop IMD. CFH, for example, is not the only regulator of the complement activation pathway. Other regulators exist that control different stages of the complement cascade and it may be the specific combination of all these factors, rather than each of them individually, to determine the fate of host-pathogen interaction following meningococcal acquisition.

## 3 *Neisseria Meningitidis* Virulence Factors

The investigation of genetic elements that could be associated to, and explain, a *Nm* pathogenic phenotype has received considerable attention in recent years [9,10]. The meningococcus is the best characterized member of the *Neisseria* genus. Following the introduction of the multilocus sequence typing system (MLST) [31] and the advent of high throughput sequencing technologies, it became possible to appreciate that *Nm* species is characterized by extensive genetic diversity and dynamic changes in DNA content and organization [32,33]. Despite this heterogeneity, however, the population is structured in groups of closely related strains, called clonal complexes [31] which, in turn, are clustered together into phylogenetic clades, a top-level population compartment [34].

Molecular epidemiology studies based on MLST typing revealed a strong association between certain bacterial lineages and invasive disease, with a minority of clonal complexes being responsible for the majority of IMD cases worldwide [35]. As an example, the sequence type 5 (ST-5) complex, represented almost exclusively by serogroup A strains, showed a disease to carriage ratio of 19.5, while the ST-8 complex, mainly represented by B and C serogroups, reached 24.5. Even within the same clonal complex, individual lineages can show different virulence levels. A meta-analysis, based on information retrieved from the pubMLST database (www.pubmlst.org), showed that the ST-41 is characterized by an increased likelihood to cause IMD compared to other members of the ST-41/44 complex. Similarly, clonal complexes can also be significantly associated with asymptomatic carriage, as is the case of ST-23, which was observed to reach disease to carriage ratios as low as <0.1 [3]. Similar disproportions were also observed in time- and population-matched strain collections [36]. The observed variance in IMD rates across different clonal complexes suggests that the ability to cause infection is mainly an intrinsic characteristic of the meningococcus and, as such, it is encoded in its genome.

Analysis of the first ever decoded genome sequence of an *Nm* isolate (strain MC58) [37] identified a list of 104 genes coding for putative virulence factors. Successively, others have been proposed through comparative pathogenomics studies or after the genomic sequencing of new *Nm* strains (a comprehensive list of meningococcal known and putative virulence factors is reported in Table 1). Association between those genes and meningococcal virulence was based on the ability of the encoded proteins to impact the bacterial surface phenotype and its interaction with the human nasopharyngeal epithelia.

**Table 1:** List of virulence factors identified in *Nm* and their association with repeat elements. Consolidated list of *Nm* virulence factors retrieved from Ampattu BJ et al. (2017) [56], Criss A et al. (2012) [57], Echenique-Rivera H et al. (2011) [58], Schoen C et al. (2006) [59], Schoen C et al. (2008) [10], Snyder L et al. (2006) [9], Tettelin H et al. (2000) [37] publications and the virulence factor database (VFDB) [60]. In the table is reported their association with repeat elements in Nm. Both known and putative virulence factors are listed. ND: no homologue detected in MC58 genome.

Virulence factor	Function	Gene symbol	*N meningitidis* MC58	Association with repeat elements
Adhesion and penetration protein	Adherence	*app*	NMB1985	Yes [15]
Adhesion	Adherence	*hsf*	NMB0992	Yes [54]
Lipooligosaccharide (LOS) sialylation	Adherence	*lst*	NMB0922	
LOS synthesis	Adherence	*kdtA*/*waaA*	NMB0014	
LOS synthesis	Adherence	*lgtA*	NMB1929	Yes [15,43]
LOS synthesis	Adherence	*lgtB*	NMB1928	
LOS synthesis	Adherence	*lgtC*	ND	Yes [43]
LOS synthesis	Adherence	*lgtE*	NMB1926	Yes [15]
LOS synthesis	Adherence	*lgtF*	NMB1704	
LOS synthesis	Adherence	*lgtG*	NMB2032	Yes [15,43]
LOS synthesis	Adherence	*lgtH*	ND	Yes [43,54]
LOS synthesis	Adherence	*rfaC*	NMB2156	
LOS synthesis	Adherence	*rfaE*	NMB0825	
LOS synthesis	Adherence	*rfaF*	NMB1527	
LOS synthesis	Adherence	*rfaK*	NMB1705	
Lipopolysaccharide (LPS) synthesis	Adherence	*lptA*	NMB1638	
LPSsynthesis	Adherence	*lpxA*	NMB0178	
LPS synthesis	Adherence	*lpxB*	NMB0199	
LPS synthesis	Adherence	*lpxC*	NMB0017	
LPS synthesis	Adherence	*lpxD*	NMB0180	
LPS synthesis	Adherence	*rfaD*	NMB0828	
Neisseria adhesion A	Adherence	*nadA*	NMB1994	Yes [15,43]
Phosphoglucomutase/LOS synthesis	Adherence	*pgm*	NMB0790	
Pilin glycosylation	Adherence	*pglA*	NMB0218	Yes [43,54]
Pilin glycosylation	Adherence	*pglB*	NMB1820	
Pilin glycosylation	Adherence	*pglC*	NMB1821	
Pilin glycosylation	Adherence	*pglD*	NMB1822	
Quinolinate synthetase	Adherence	*NEIS1772*	NMB0394	
Type IV pili	Adherence	*pilC*	NMB0049	Yes [15,43,54,55]
Type IV pili	Adherence	*pilD*	NMB0332	
Type IV pili	Adherence	*pilE*	NMB0018	
Type IV pili	Adherence	*pilF*	NMB0329	
Type IV pili	Adherence	*pilG*	NMB0333	
Type IV pili	Adherence	*pilH*	NMB0886	
Type IV pili	Adherence	*pilI*	NMB0887	
Type IV pili	Adherence	*pilJ*	NMB0888	
Type IV pili	Adherence	*pilK*	NMB0889	
Type IV pili	Adherence	*pilM*	NMB1808	
Type IV pili	Adherence	*pilN*	NMB1809	
Type IV pili	Adherence	*pilO*	NMB1810	
Type IV pili	Adherence	*pilP*	NMB1811	
Type IV pili	Adherence	*pilQ*	NMB1812	Yes [15]
Type IV pili	Adherence	*pilS*	NMB0020	Yes [15]
Type IV pili	Adherence	*pilT2*	NMB0768	
Type IV pili	Adherence	*pilT*	NMB0052	
Type IV pili	Adherence	*pilU*	NMB0051	
Type IV pili	Adherence	*pilV*	NMB0547	
Type IV pili	Adherence	*pilW*	NMB1309	
Type IV pili	Adherence	*pilX*	NMB0890	Yes [43,54]
Type IV pili	Adherence	*pilZ*	NMB0770	
Lactate permease	Colonization	*lctP*	NMB0543	
Lipoprotein NlpD	Colonization	*NEIS1418*	NMB1483	
FarAB	Efflux pump	*farA*	NMB0318	
FarAB	Efflux pump	*farB*	NMB0319	
MtrCDE	Efflux pump	*mtrC*	NMB1716	Yes [15]
MtrCDE	Efflux pump	*mtrD*	NMB1715	
MtrCDE	Efflux pump	*mtrE*	NMB1714	
Capsule	Immune evasion	*ctrA*	NMB0071	
Capsule	Immune evasion	*ctrB*	NMB0072	
Capsule	Immune evasion	*ctrC*	NMB0073	
Capsule	Immune evasion	*ctrD*	NMB0074	
Capsule	Immune evasion	*ctrG*	NMB0065	
Capsule	Immune evasion	*lipA*	NMB0082	
Capsule	Immune evasion	*lipB*	NMB0083	
Capsule	Immune evasion	*mynA*/*sacA*	ND	
Capsule	Immune evasion	*mynB*/*sacB*	ND	
Capsule	Immune evasion	*mynC*/*sacC*	ND	
Capsule	Immune evasion	*mynD*/*sacD*	ND	
Capsule	Immune evasion	*siaA*/*synA*	NMB0070	
Capsule	Immune evasion	*siaB*/*synB*	NMB0069	
Capsule	Immune evasion	*siaC*/*synC*	NMB0068	
Capsule	Immune evasion	*siaD*/*synD*	NMB0067	Yes [15,43]
Capsule	Immune evasion	*synE*	ND	
Drug resistance	Immune evasion	*ermE*	NMB0393	Yes [15]
Protease	Immune evasion	*NEIS2103*	NMB2127	
T-cell stimulating protein	Immune evasion	*tspB*	NMB1548	
Factor H binding protein	Immune modulator	*fHbp*	NMB1870	
Neisserial surface protein A	Immune modulator	*nspA*	NMB0663	
Class 5 outer membrane protein	Invasion	*opc*	NMB1053	Yes [15,43,54]
Other outer membrane proteins	Invasion	*rmpM*	NMB0382	Yes [15]
Other outer membrane proteins	Invasion	*mlp*	NMB1898	
Other outer membrane proteins	Invasion	*Omp85*	NMB0182	
Other outer membrane proteins	Invasion	*OmpH*	NMB0181	
Other outer membrane proteins	Invasion	*NEIS1917*	NMB1946	
Regulation of capsule expression	Invasion	*misS*/*phoQ*	NMB0594	
Regulation of capsule expression	Invasion	*misR*/*phoP*	NMB0595	
Type I secretion protein	Invasion	*tolC*	NMB1737	
VacJ-related protein	Invasion	*NEIS1933*	NMB1961	
Opacity protein	Invasion	*opa*	NMB0442	Yes [15,43,54]
PorA	Invasion	*porA*	NMB1429	Yes [15,43,54]
PORB	Invasion	*PORB*	NMB2039	Yes [43,54]
Infectivity potentiator	Invasion	*NEIS0982*	NMB0995	
Infectivity potentiator	Invasion	*NEIS1487*	NMB1567	
ABC transporter	Iron uptake systems	*fbpA*	NMB0634	
ABC transporter	Iron uptake systems	*fbpB*	NMB0633	
ABC transporter	Iron uptake systems	*fbpC*	NMB0632	
ABC transporter	Iron uptake systems	*NEIS1964*	NMB1989	
ABC transporter	Iron uptake systems	*NEIS1965*	NMB1990	
ABC transporter	Iron uptake systems	*NEIS1966*	NMB1991	
ABC transporter	Iron uptake systems	*fetB2*	NMB1880	
Bacterioferritin	Iron uptake systems	*bfrA*	NMB1207	
Bacterioferritin	Iron uptake systems	*bfrB*	NMB1206	
Bacterioferritin	Iron uptake systems	*bcp*	NMB0750	
Control of iron homeostasis genes	Iron uptake systems	*fur*	NMB0205	
Ferric enterobactin transport protein A/ferric-repressed protein B	Iron uptake systems	*fetA*/*frpB*	NMB1988	Yes [15,43,54]
Ferrochelatase	Iron uptake systems	*hemH*	NMB0718	
Hemoglobin receptor	Iron uptake systems	*hmbR*	NMB1668	Yes [15,43,54]
Hemagglutinin/hemolysin	Iron uptake systems		NMB0493	
Hemagglutinin/hemolysin	Iron uptake systems		NMB0497	
Hemagglutinin/hemolysin	Iron uptake systems		NMB1214	
Hemagglutinin/hemolysin	Iron uptake systems		NMB1779	
Heme uptake	Iron uptake systems	*hpuA*	ND	Yes [43,54]
Heme uptake	Iron uptake systems	*hpuB*	ND	
Hemolysin	Iron uptake systems		NMB0496	
Hemolysin	Iron uptake systems	*NEIS1560*	NMB1646	
Hemolysin activator	Iron uptake systems	*NEIS1658*	NMB1738	
Hemolysin activator	Iron uptake systems	*tpsB*	NMB1780	
Iron uptake system component	Iron uptake systems	*NEIS0012*	NMB0035	
Lactoferrin-binding protein	Iron uptake systems	*lbpA*	NMB1540	Yes [43]
Lactoferrin-binding protein	Iron uptake systems	*lbpB*	NMB1541	Yes [15,43,54]
Ton system	Iron uptake systems	*exbB*	NMB1729	
Ton system	Iron uptake systems	*exbD*	NMB1728	
Ton system	Iron uptake systems	*NEIS1887* (*fhuA*)	NMB0293	
Ton system	Iron uptake systems	*NEIS1282*	NMB1346	
Ton system	Iron uptake systems	*NEIS2529*	NMB1449	Yes [15]
Ton system	Iron uptake systems	*NEIS0387*	NMB1829	
Ton system	Iron uptake systems	*NEIS0338*	NMB1882	
Ton system	Iron uptake systems	*tonB*	NMB1730	
Transferrin-binding protein	Iron uptake systems	*tbpA*	NMB0461	Yes [54]
Transferrin-binding protein	Iron uptake systems	*tbpB*	NMB0460	Yes [15,43,54]
Transferrin-binding protein	Iron uptake systems	*NHBA*	NMB2132	Yes [55]
3R-hydroxymyristoyl ACP dehydrase	Other	*fabZ*	NMB0179	
Carboxyl-terminal processing protease	Other	*prc*	NMB1332	
Hypohetical protein	Other	*NEIS0695*	NMB0741	
Hypohetical protein	Other	*NEIS0436*	NMB1786	
Hypohetical protein	Other	*NEIS1028*	NMB1064	
Nitric oxide reductase	Other	*norB*	NMB1622	
Nucleotides metabolism	Other		NMB0757	
Putative integral membrane protein	Other	*NEIS0377*	NMB1840	
Serine protease	Other	*nalP*	NMB1969	Yes [54]
Transcriptional regulator	Other	*mtrR*	NMB1717	
Uncharacterized protein	Other		NMB1828	
VapD-like protein	Other		NMB1753	
IgA protease	Stress response	*iga*	NMB0700	Yes [15,54]
Iron-sulphur protein	Stress response	*NEIS1371*	NMB1436	
Iron-sulphur protein	Stress response	*NEIS1372*	NMB1437	
Iron-sulphur protein	Stress response	*NEIS1373*	NMB1438	
Catalase	Stress response	*katA*	NMB0216	
Endonuclease	Stress response	*nth*	NMB0533	
Manganese transport system	Stress response	*mntA*	NMB0588	
Manganese transport system	Stress response	*mntB*	NMB0587	
Manganese transport system	Stress response	*mntC*	NMB0586	
Methionine sulphoxide reductase	Stress response	*msrA*/*B*(*pilB*)	NMB0044	
Nitrite reductase	Stress response	*pan1*	NMB1623	
Recombinational repair protein	Stress response	*recN*	NMB0740	Yes [15]
Superoxide dismutase	Stress response	*sodB*	NMB0884	
Superoxide dismutase	Stress response	*sodC*	NMB1398	
FrpC operon protein	Toxin		NMB0364	
FrpC operon protein	Toxin		NMB0365	
FrpC operon protein	Toxin		NMB0584	
FrpC operon protein	Toxin		NMB1409	
FrpC operon protein	Toxin		NMB1412	
FrpC operon protein	Toxin		NMB1414	
Neisseria ADP-ribosylating enzyme	Toxin	*narE*	NMB1343	
Putative toxin-activating protein	Toxin		NMB1210	
Putative toxin-activating protein	Toxin		NMB1763	
RTX toxin	Toxin	*frpA*	NMB0585	
RTX toxin	Toxin	*frpC*	NMB1415	Yes [54]
Oxidoreductase	Stress protein	*dsbA*-*1*	NMB0278	
Oxidoreductase	Stress protein	*dsbA*-*2*	NMB0294	
Oxidoreductase	Stress protein	*dsbA*-*3*	NMB0407	

As additional genomic sequences became available, various attempts to characterize the genetic elements associated with an invasive phenotype were made. These focused both on the exploration of nucleotide sequence variation at shared loci and on the variation in the gene content. Comparisons of the meningococcal gene repertoire with those of other, less pathogenic, *Neisseria* species failed to identify consistent differences. Moreover, despite the different trophism of human colonization, *Nm* was found to share most of its genetic content with *N*. *lactamica* and *N*. *gonorrhoeae* [9,38]. Similarly, genome wide association studies comparing pathogenic and apathogenic strains could not reveal unambiguous evidences of the presence of indispensable virulence factors [9,10]. The capsule region, containing clusters of genes encoding the ability to synthesize the polysaccharide layer, has been regarded as the main meningococcal virulence determinant, given the fact that 5 (A, B, C, W and Y) of the 12 serogroups known to date are responsible for the vast majority of IMD cases [7]. Additionally, a putative phage element was found to be significantly associated with meningococcal disease. Despite the strong association however, more that 50% of healthy carriers in the analyzed population were colonized with an *Nm* isolate carrying the phage element within their genome [39]. Overall, collected evidences indicate that the propensity to cause disease is a multifactorial property, which depends on combinations of genes and genetic elements that, individually, are commonly found also in non-pathogenic lineages.

## 4 *Neisseria Meningitidis* Genome Variability Factors

*Nm*, like other obligate commensals, must face several hurdles in order to successfully colonize a genetically and immunologically diverse host population. During meningococcal transmission, only a small minority of colonizing cells is likely to be transmitted to the new host. In the peculiar environmental settings provided by the new hosting organism, newly transmitted cells must be able to adhere to endothelial cells while also scavenging nutrients and avoid host's defense mechanisms. It is postulated that the highly mutable genome characterizing the meningococcal species has evolved in response to the need to survive in such a dynamic environment. The ability to quickly generate many different phenotypes, in fact, allows for the exploration of alternative phenotypic solutions from which the fittest can be selected for survival and subsequent transmission [40,41].

Based on this theory, it would be intuitive to expect a positive selection for an increased mutation rate in bacterial species that are subjected to major environmental fluctuations. However, deleterious mutations have a higher chance to occur compared to beneficial ones and a generalized increase in genome mutability would inevitably result in an evolutionary dead-end. Presumably to meet this challenge, organisms like *Nm* have evolved strategies to focus high mutation rates in those genes that are involved in critical interactions with the host, without increasing the overall mutability of their genome [40,42]. Since the first *Nm* genome sequences became available it soon became evident that this species have accumulated thousands of repetitive sequence elements in its genome, ranging from basic homopolymeric tandem repeats to complete gene clusters duplications [43]. The different types of repetitive elements, which are listed in Table 2, function as variability hotspots as they can be prone to slipped strand mispairing during chromosomal replication, promote the uptake of exogenous DNA or function as hotspots for chromosomal rearrangements. It has been proposed that the coexistence within bacterial genomes of such “contingency” chromosomic regions and more stable “housekeeping” regions could facilitate the efficient exploration of phenotypic solutions to unpredictable aspects of the host environment, while minimizing deleterious effects on bacterial fitness [40,41]. Several putative virulence genes have been reported to be associated with one or more of these repeat elements (Table 1).

**Table 2:** Families of repeat elements characterizing the *Nm* genome.

Repeat element	Composition	Putative function	Reference
ATR (AT-rich repeats)	183-bp A + T-rich sequence whose ends form an imperfect 35-bp inverted repeat	Modulation of gene expression	Parkhill J et al., Nature (2010) and Ampattu BJ et al., (2017)
Coding tandem repeats	Tandem repeats that do not disrupt the reading frame (repeat unit composed of 3 bp or multiples of 3 bp)	Generation of differing protein isoforms	Jordan P et al., BMC Microbiol (2003)
CREE (Correia repeat enclosed elements)	156-bp sequence bounded by a 26-bp inverted repeat	Modulation of gene expression	Correia FF et al., J Biol Chem (1988)
DUS (DNA uptake sequence)	10-bp sequence 5′-GCCGTCTGAA-3′	Recognition and uptake of exogenous DNA	Goodman SD and Scocca JJ, Proc Natl Acad Sci USA (1988)
NIME (neisserial intergenic mosaic elements)	Repeat units of 50–150 bp (RS elements), each flanked by 20-bp inverted repeats (dRS3 elements)	Pilin genes recombination	Parkhill J et al., Nature (2010)
SSR (simple sequence repeats)	1- to 10-bp motifs that are repeated in tandem	Modulation of gene expression	Saunders NJ et al., Mol Mircobiol (2000)
REP 2	120–150 bp sequence containing ribosome-binding-site-like conserved AAGGA motif	Modulation of gene expression	Parkhill J et a.l, Nature (2010)
REP 3	60-bp conserved sequence occurring next to CREE elements	Unknown	Parkhill J et al., Nature (2010)
REP 4	26-bp conserved sequence occurring next to CREE elements	Unknown	Parkhill J et al., Nature (2010)
REP 5	20-bp conserved sequence occurring next to CREE elements	Unknown	Parkhill J et al., Nature (2010)

**Fig. 1:**
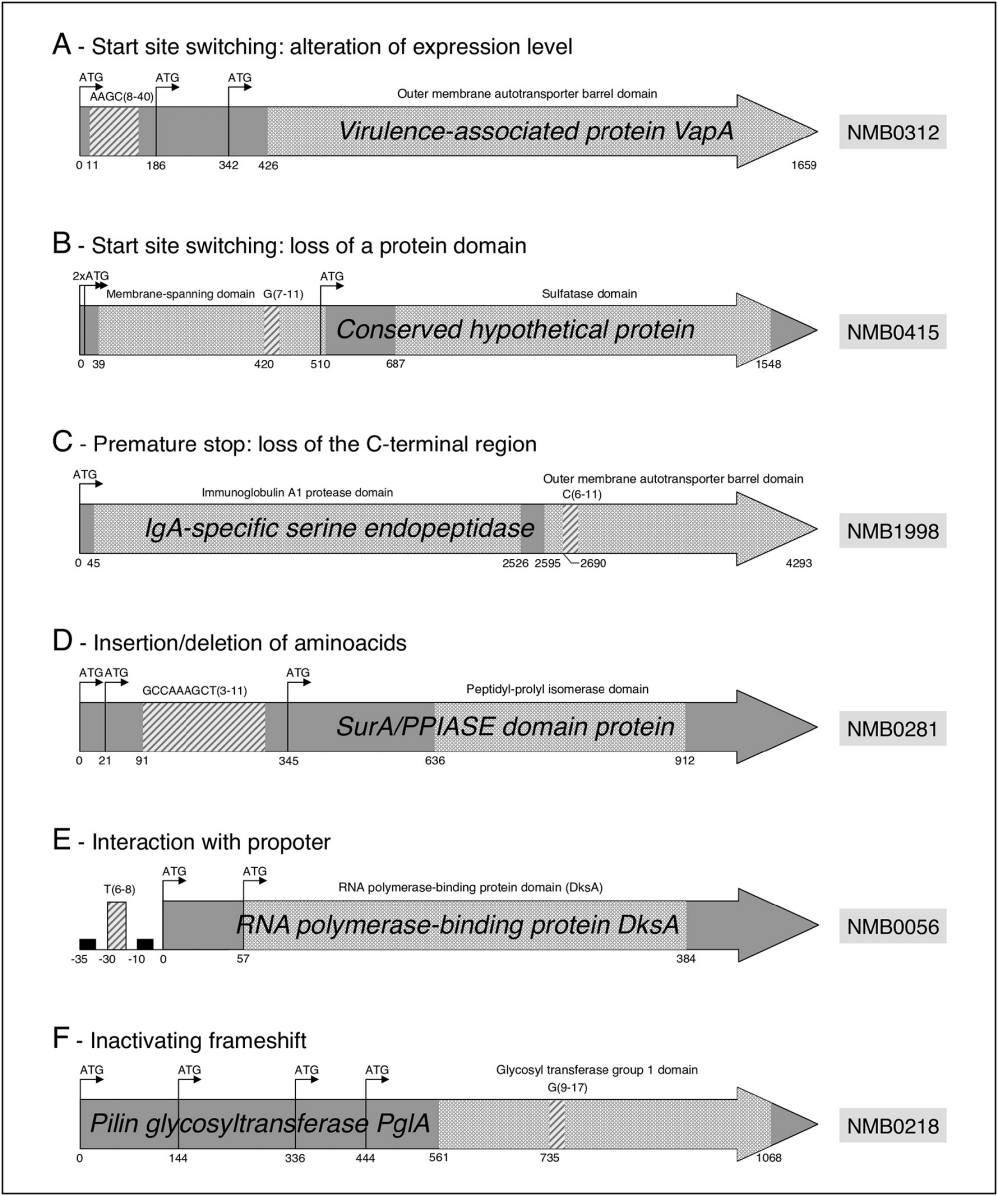
Schematic representation of putative SSRs modes of action. A: Variable number simple sequence repeat (VNSSR) causing translational start site switching. B: VNSSR causing the loss of a membrane-spanning domain. C: VNSSR leading to the loss of the peptide C-terminal region. D: VNSSR introducing changes in the peptide sequence. E: VNSSR influencing the gene promoter. F: VNSSR introducing an inactivating frame shift. Dark grey arrows represent open reading frames. Black arrows marked with ATG represent in-frame ATG translational start sites. Light grey boxes represent the annotated functional domains. Stripped boxes represent VNSSRs and the related tags indicate the repeat unit motif along with the minimum and maximum number of repetitions observed in the 20 analyzed genomes. Numbers below each gene indicate the position relative to the annotated translational tart site. Reproduced from Siena et al. [15].

A major contribution to *Nm* genotypic variability is provided by SSRs, extended stretches of repeated nucleotide motifs that are highly prone to replication errors [17]. SSRs located within gene coding sequences or in the proximity of their promoters can either modulate the level of gene expression or produce alternate protein variants through a number of mechanisms [41,44,45] (Fig. 1). A recent comparative genomic study performed by our group highlighted an unappreciated potential for SSR-mediated phase variation to promote phenotypic variation [15]. Each meningococcal strain was found to contain an average of 4243 SSRs in its genome, which if normalized for the typical chromosome size (≈2.2 million nucleotides) account for the extraordinary SSRs density of one repeat every 520 nucleotides. This enrichment for SSRs in *Nm* was found to be unusually high compared to other prokaryotes [16] or random expectation [17]. Subsequent *in vitro* testing allowed to appreciate that a substantial portion of these SSRs underwent length polymorphisms in strains grown overnight in non-selective conditions. Within this short time frame, these SSRs element could destabilize the chromosomic regions related to 115 different genes, possibly leading to a modulation of their expression or complete silencing. Even in the simplest case of an on/off type of regulation, the random combinatorial switching of these 115 contingency genes could already produce an enormous amount of alternative phenotypes (2^115^). In line with the aforementioned within-host evolution theory, these genes are enriched for cell surface determinants relevant to bacteria-host interaction [15].

## 5 Interplay between Virulence and Variability Factors in Invasive Meningococcal Disease

A recent study conducted by Klughammer et al. [11] investigated the within-host genetic changes occurring in meningococcus by comparing the genomic sequences of throat-blood isolate pairs from four patients suffering from acute IMD. Even if based on a limited number of cases, this study showed that strains that could penetrate the nasopharyngeal epithelium (*i*.*e*. pathogenic strains) were characterized by mutations predominantly affecting the biogenesis of the meningococcal type IV pilus, a main surface determinant. Not a single set of mutations was shared by all the analyzed strain pairs, underlying the stochastic nature of these events. Moreover, mutations were primarily contributed by the variability factors described above, 8 (73%) of which were represented by length polymorphisms occurring at SSRs sites. Even though the association between genetic elements capable of promoting phenotypic variation and pathogenic traits has been hypothesized long ago [40], this study represents the first experimental confirmation.

**Fig. 2:**
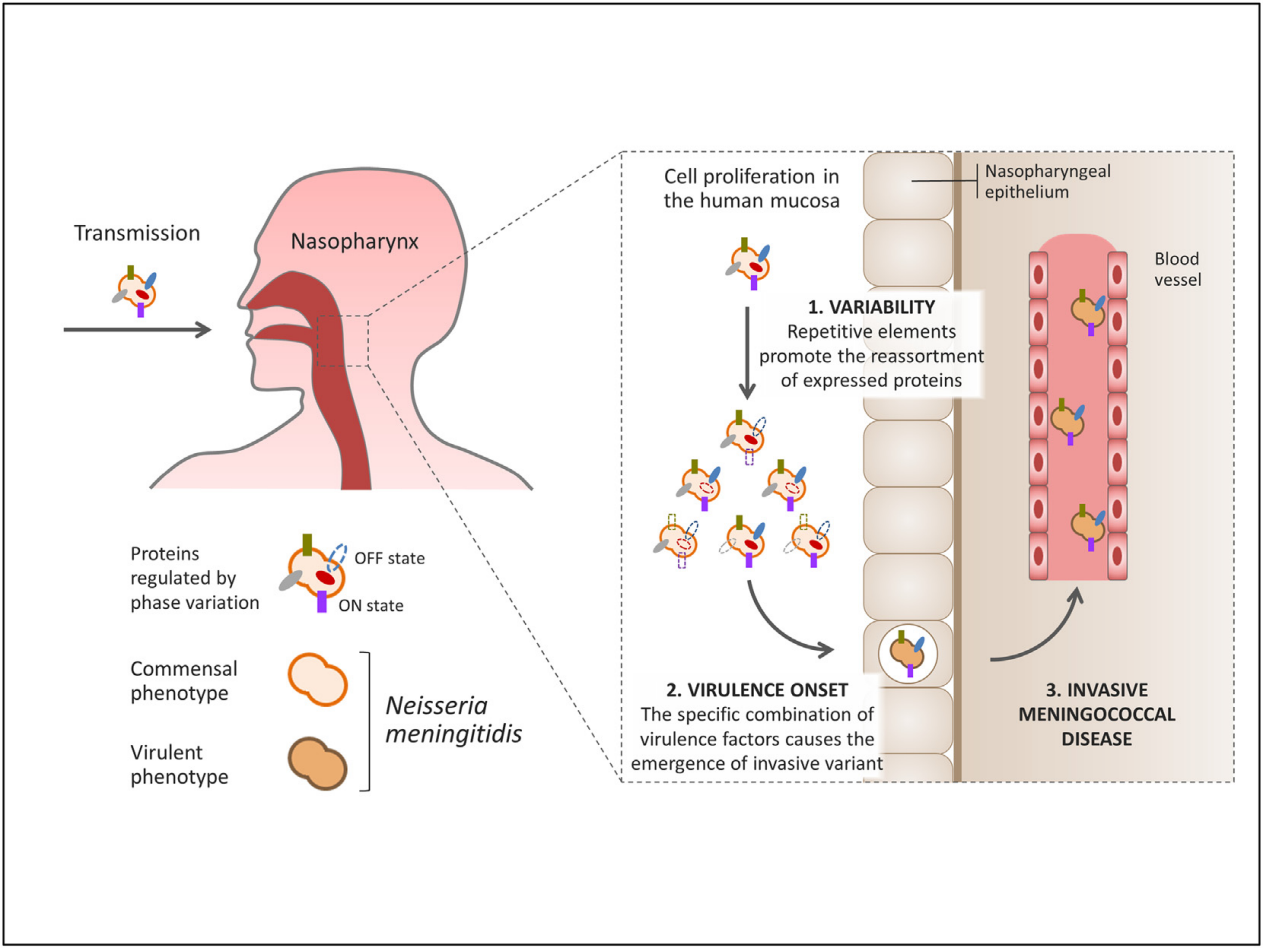
Proposed model for the onset of invasive meningococcal disease. Following transmission and colonization of the human nasopharynx, the founder clone starts proliferating. During this phase, extensive phenotypic variation is generated by the stochastic reassortment of virulence factors (surface determinants and genes involved in host-pathogen interaction) driven by meningococcal chromosomic variability factors (step 1). This exploration of new phenotypic solutions can lead to the accidental onset of a virulent variant (step 2), which is able to penetrate the nasopharyngeal epithelial barrier and cause septicemia (step 3).

Meningococcal disease has been proposed to occur within few days after the acquisition of a new *Nm* isolate in the nasopharynx [1,46]. This fast-track from acquisition to invasive disease is compatible with the short time required by SSRs to modulate gene expression and promote phenotypic variation. Evidences collected by Klughammer et al. confirm that length polymorphisms at SSR loci are indeed capable of generating the genetic diversity observed in the throat-blood isolate pairs during nasopharyngeal carriage and suggest that IMD likely results of the within-host evolution of the colonizing isolate, which is driven by the specific interplay between virulence and variability factors. A cartoon summarizing this process is shown in Fig. 2. According to this hypothesis, only one, or a limited number of bacterial cells are successfully transmitted to the new host. After colonization of the human mucosa, the founder cells start proliferating while also trying to increase their fitness by exploring alternative phenotypic solutions, which are generated by SSRs and similar variability factors. During this process, chances are that the random reassortment of proteins relevant to the interaction with the host would produce a pathogenic variant capable of crossing the nasopharyngeal epithelium, access the bloodstream and cause systemic infection.

As a further support to this hypothesis is the fact that the associations between virulence and variability factors characterized to date (Table 1) almost exclusively involve genes coding for proteins that are involved in the interaction with the host and that are located to the cell outer membrane and, as such, are potential targets of hosts immune defense mechanisms. These can be broadly categorized into evasins, adhesins, lipopolysaccharide (LPS) biosynthesis and iron acquisition proteins.

Evasins are a family of proteins whose function is to help escaping the host immune defenses. Capsular polysaccharides constitute a barrier that enables bacteria to resist phagocytosis and complement mediated killing. In *Nm* the capsule production is controlled by a peptide encoded by *siaD*, a gene whose expression is controlled by transcriptional slippage of an intragenic homopolymeric tract [15,43].

Adhesins are a family of proteins involved in *Nm* adherence to the human epithelium and in tissue trophism. Due to the cell surface localization of these proteins, most adhesins induce antibody responses during natural infection. Opacity proteins provide an example of such function in *Nm* and for some of them the expression was found to be regulated by variable sequence repeats, like is the case of *opa* and *opc* [43,47].

LPS is a major constituent of the outer surface of Gram-negative bacteria and is intimately involved in every stage of *Nm* interaction with its host. Among the functions mediated by the LPS layer are attachment of bacterial cells to host membranes and resistance to the innate immune system. Seven *lgt* genes (*lgtA*, *lgtB*, *lgtC*, *lgtE*, *lgtF*, *lgtG* and *lgtH*), encoding for glycosyltransferases, act in different combinations to generate alternative LPS structures in *Nm*. As reported in Table 1, five of these genes are under the stochastic control of repeat elements.

Finally, one of the needs of most pathogenic bacteria is to scavenge resources from the external environment. *Nm*, for example, relies on exogenous acquisition of iron in order to maintain its fitness [48]; a need that induced this pathogen to develop alternative and partially redundant mechanisms for iron scavenging [49]. These involve numerous surface-expressed proteins that are targeted by the human immune system [47,50]. Phase variation of these loci can therefore result in antigenic variation similar to that proposed for the O*pa* genes, with deep implication in the establishment of IMD.

## 6 Summary and Outlook

Overall, several attempts were made to better understand *Nm* biology and unravel the mechanisms leading to IMD. Different host factors have been associated to altered levels of susceptibility to meningococcal infection, however none of them can accurately predict whether a given subject will develop IMD or not. Similarly, no genetic factors have been identified in *Nm* that could clearly and unequivocally distinguish between pathogenic and harmless *Nm* isolates. Nonetheless, recent findings seem to suggest that the coexistence and interaction between genetic “variability” factors, capable of increasing the mutability of specific chromosomic regions, and “virulence” factors, encoding for bacterial-host interaction functions, is likely the key trigger of *Nm* pathogenicity. This multifactorial nature of IMD is further complicated by *Nm* living within a dynamic and diverse host population, characterized by different levels of pre-existing immunity and different susceptibility to meningococcal disease. This introduces an additional layer of complexity and greatly expands the space of variables to be accounted for. A further, practical challenge comes from the difficulty to obtain blood-throat isolate pairs to be used in comparative studies, due to the low IMD incidence and immediate antibiotic treatment of hospitalized patients.

In conclusion, much progress has been made in understanding the mechanisms underlying the origin of IMD. In this regard, the interplay between “virulence” and “variability” factors is emerging as a key driver of the transition from a commensal to virulent *Nm* phenotype. Despite this, however, challenges like the complexity of *Nm* pathogenesis and the difficulties in data collection are still preventing from reconstructing the whole picture. There is little doubt that the road to understanding the origin of IMD will necessarily go through large-scale genomic comparisons of commensal and virulent *Nm* strains; *de facto* following the direction set by Klughammer and coworkers [11]. These will likely be facilitated by the most recent sequencing technologies, which allow for the characterization and study of bacterial isolates directly from clinical samples, like blood or cerebrospinal fluid [[51], [52], [53]]. In our vision, these studies will be foundational to advance our understanding of the origin of IMD.

## Conflict of Interest

This work was sponsored by GlaxoSmithKline Biologicals SA. ES and DM are employees of the GSK group of companies. MB is an employee of Randstad Italia spa, working as a contractor for GSK. ES is listed as an inventor on a patent on meningococcal polypeptide sequences, owned by the GSK group of companies. The authors report no additional conflicts of interest.

## Authors Contribution

ES and MB drafted the manuscript. DM provided intellectual input. All authors approved the final manuscript.
